# The impact of bioinformatic choices on *Coccidioides* variant identification accuracy

**DOI:** 10.1128/spectrum.01232-25

**Published:** 2025-08-15

**Authors:** Marco Marchetti, Emanuel M. Fonseca, Kimberly E. Hanson, Bridget Barker, Katharine S. Walter

**Affiliations:** 1 Eccles Institute of Human Genetics, University of Utah 208371 , Salt Lake City, Utah, USA; 2 Division of Epidemiology, University of Utah 208352, Salt Lake City, Utah, USA; 3 Elfa Analytics, Ponte Nova, Minas Gerais, Brazil; 4 Department of Medicine, Division of Infectious Diseases, University of Utah School of Medicine 12348, Salt Lake City, Utah, USA; 5 Department of Pathology, Division of Clinical Microbiology, University of Utah and ARUP Laboratories 161530, Salt Lake City, Utah, USA; 6 Department of Biological Sciences, Northern Arizona University 3356, Flagstaff, Arizona, USA; Brown University, Providence, Rhode Island, USA

**Keywords:** *Coccidioides posadasii*, *Coccidioides immitis*, fungal genomics, repetitive elements, species identification, variant calling pipeline

## Abstract

Emerging fungal pathogens, such as *Coccidioides*, the causative agent of Valley fever, or coccidioidomycosis, pose significant clinical and public health challenges. While advances in genomic epidemiology have enhanced our understanding of *Coccidioides* evolutionary history, effective variant identification is complicated by the genome’s structural complexity. Repetitive elements, transposable sequences, and regions of low complexity can lead to incorrect variant calls, affecting downstream analyses. Further, accurate species identification is essential for understanding the spread of *C. immitis* and *C. posadasii*, which, despite having distinct primary geographic distributions, can co-occur. Distinguishing between these species is critical for interpreting patterns of transmission, emergence, and potential shifts in endemicity. To address this, we developed a pipeline to identify genetic variants and assign species directly from sequencing reads. We evaluated the performance of variant identification both across the genome and after excluding repetitive regions identified by NUCmer, a commonly used tool, on simulated genomic data and empirically generated sequence data. Whole-genome calling detected the highest number of single-nucleotide polymorphisms (SNPs), over 80,000 on average in both species, but included a substantial number of false positives, with 42,834 true positives and 38,115 false positives identified. Masking repetitive regions significantly enhanced accuracy. In *C. immitis*, masking with NUCmer increased sensitivity from 70.1% to 91.7% and precision from 52.7% to 91.1%. Similarly, in *C. posadasii*, sensitivity improved from 80.0% to 96.1% and precision from 53.1% to 90.4%. These improvements were also reflected in overall F1 scores, which rose from 60%–64% in whole-genome analysis to over 90% after masking. Using simulated reads, our pipeline recovered 83,400 SNPs in *C. posadasii*, with 40,163 shared across regions and a Jaccard index of 0.36. Species classification was highly accurate—100% in simulations and 98.9% in 175 publicly available samples. Here, we provide a benchmarked variant and species identification pipeline for *Coccidioides* and quantify the impact of genomic region on variant identification performance, which may have downstream impacts on phylogenetic and genomic epidemiology inference.

**IMPORTANCE:**

Accurate genetic analysis is essential for tracking and understanding emerging fungal pathogens like *Coccidioides*, the cause of Valley fever. However, the complex structure of fungal genomes makes it difficult to identify genetic differences reliably. This study demonstrates that the choice of genomic regions has a substantial impact on variant detection accuracy. We developed and tested a new tool called *cocci-call* and found that focusing on specific regions of the genome dramatically improves the accuracy of genetic variant detection. This improvement could enhance how researchers monitor outbreaks, track fungal evolution, and design better diagnostics. By identifying high-confidence regions for analysis, our work helps standardize how *Coccidioides* genomes are studied and compared, laying the groundwork for more accurate and reproducible genomic research in this important pathogen.

## INTRODUCTION

Emerging fungal pathogens are an escalating public health threat, demanding urgent attention from the global health community (1). In 2022, the World Health Organization (WHO) underscored this urgency by publishing its first-ever list of fungal pathogens prioritized for public health research, placing *Coccidioides* spp., the causative agents of coccidioidomycosis or Valley Fever, among the most critical (2). Valley Fever is caused by two closely related fungal species, *Coccidioides immitis* and *C. posadasii*, which exhibit distinct geographical distributions. *Coccidioides immitis* predominantly occurs in California, Baja California (Mexico), and Washington state, while *C. posadasii* is more widespread, spanning Arizona, New Mexico, Texas, and various regions of Latin America (3). Given their clinical significance, genomic epidemiology has become a crucial tool for understanding *Coccidioides* transmission, genetic diversity, and evolutionary dynamics (4–6).

However, the utility of genomic data in these efforts depends heavily on the accuracy of variant identification. Many genomes, including those of *Coccidioides*, contain repetitive elements, transposable sequences, and regions of low complexity that can lead to misaligned reads and spurious variant calls (7, 8). Variants detected in these regions may not represent true genetic differences but rather artifacts caused by sequencing biases or errors in read mapping. Ignoring these complexities can introduce inaccuracies that compromise downstream analyses, such as phylogenetic and population genetics inference.

To address this challenge, “high-confidence” genomic regions have been identified in the human genome. Variants within these high-confidence regions are often the focus of bioinformatic tool benchmarking and included in genomic analyses (9–11). However, such high-confidence regions have not been identified in *Coccidioides,* nor has the accuracy of variant identification within and outside these regions been measured. The choice of high-confidence region is impacted by tradeoffs in sensitivity and specificity of variants. While including variation across the whole genome may maximize the number of variants detected, they may also include regions prone to high false-positive rates. Conversely, filtering out certain regions, such as those identified as repetitive, may improve precision but at the cost of excluding potentially meaningful variants.

To provide a tool for reproducible, benchmarked genomic variant analysis in *Coccidioides*, we developed a bioinformatic pipeline for *Coccidioides* variant identification and species assignment. We benchmarked variant identification across different genomic regions.

## MATERIALS AND METHODS

### Variant calling and species identification in *Coccidioides*

To ensure high-quality variant calling in *Coccidioides*, we implemented an automated preprocessing and filtering pipeline to remove low-quality reads, eliminate potential contamination, and assign reads to species before downstream analysis. The pipeline was developed and benchmarked using Illumina short-read sequencing data, which is the predominant platform used in *Coccidioides* genomics but is compatible with other comparable sequencing platforms. First, raw reads were preprocessed by trimming low-quality bases (Phred-scaled base quality <20) and removing adapters using Trim Galore v. 0.6.10 (stringency = 1). Quality control reports were then generated with FastQC. To minimize contamination from other species, a potential source of false variant calls, Kraken2 (12) was used to taxonomically classify reads and remove those not assigned to the *Coccidioides* genus. Species assignment was determined using a log_2_-transformed ratio of Kraken2 unique minimizers mapped to *C. immitis* and *C. posadasii*. Regardless of species assignment, all *Coccidioides* reads were retained for downstream analysis, as hybridization between the two sister species has been documented and represents a potential source of true biological variation (8). Next, reads were mapped to two reference genomes, GCA_000149335.2 for *C. immitis* and GCA_018416015.2 for *C. posadasii*, using BWA v. 0.7.17 (*bwa mem*) (13). The appropriate genome is selected based on the classification from the previous Kraken2 step. For samples where the log_2_-transformed ratio of Kraken2 unique minimizers fell below the threshold of 1.5, reads were mapped to both reference genomes, and species assignment was based on the highest mapping percentage. Duplicate reads were marked with GATK 4.3 (14). Variants were called using GATK 4.3 HaplotypeCaller and GenotypeGVCFs, with sample ploidy set to 1. After variant calling, VCF files were filtered to retain single-nucleotide polymorphisms (SNPs) with a minimum depth of 10× and a minimum variant quality score of 20 (Phred scale). These thresholds were selected to reduce the probability of false-positive calls, as a Phred score of 20 corresponds to a base call accuracy of 99% (1% error rate). Because *Coccidioides* is haploid, all observed allelic variation at a site is assumed to result from sequencing or alignment error. A minimum depth of 10× provides sufficient support to distinguish true variants from sequencing error. Additionally, variants with depth or coverage deviating more than two standard deviations from the mean were removed, as they frequently occurred in repetitive regions when using unmasked reference genomes. Finally, filtered variants were annotated using SnpEff (15) using databases generated from the same assemblies used for alignment and variant calling, and a consensus FASTA sequence was generated with bcftools v. 1.17 (16).

**Fig 1:**
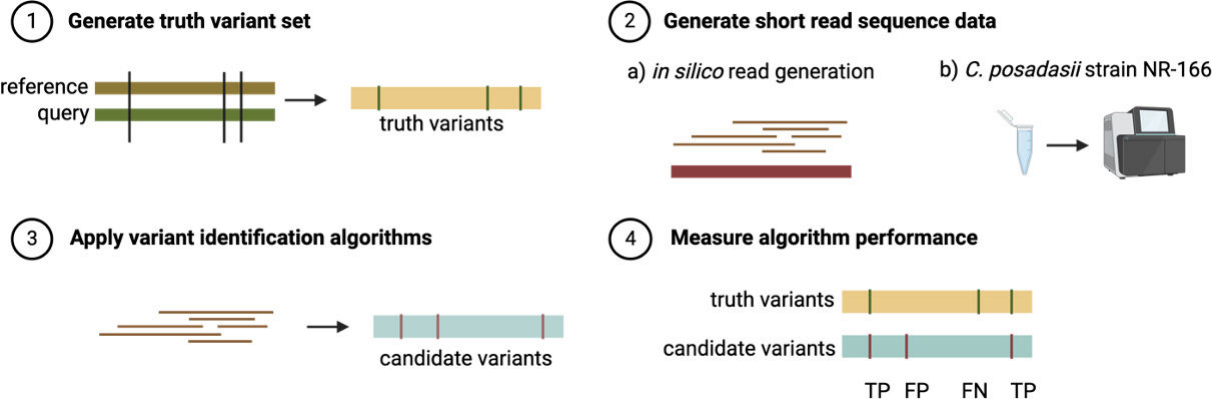
Workflow for benchmarking variant identification algorithms in *Coccidioides* species. Step 1: Generate a truth variant set. The true set of genetic variants is identified by comparing a reference genome with a query genome. This comparison produces a set of “truth variants” that serve as a benchmark. Step 2: Generate short-read sequence data. Short-read sequence data are generated in two ways: (a) *in silico* read generation, where simulated reads are computationally generated from the query genome; and (b) sequencing of *C. posadasii* strain NR-166, where biological reads are obtained experimentally. Step 3: Apply variant identification algorithms. The generated sequence reads are processed through variant-calling algorithms, producing a set of “candidate variants” that can be compared with the truth variant set. Step 4: Measure algorithm performance. The accuracy of the variant-calling algorithm is assessed by comparing the truth variant set to the candidate variants, with performance metrics calculated based on true positives (TP), false positives (FP), and false negatives (FN).

An integral feature of our pipeline is the early species identification step, where raw sequencing reads are taxonomically classified using Kraken2. This classification informs subsequent mapping, with reads assigned to *C. immitis* or *C. posadasii* aligned only to the corresponding reference genome. In addition to variant calling, we assessed the accuracy of this species assignment approach and found it to be highly reliable. To promote reproducibility and consistency across studies, we developed a standardized and freely accessible pipeline designed to streamline genome-wide variant detection and species identification in *Coccidioides*. We call it cocci-call, and it is available on GitHub (https://github.com/ksw9/cocci-call). cocci-call is structured to efficiently process large sequencing data sets while maintaining flexibility for diverse research applications. The pipeline was developed using the Nextflow workflow language, enabling scalable execution on high-performance computing (HPC) clusters. Moreover, the SLURM job scheduler is leveraged to execute tasks, allowing for multiple samples to be analyzed in parallel rather than sequentially. cocci-call employs Docker containers to ensure reproducibility across different computational environments. Hereafter, we refer to our variant identification pipeline as cocci-call (Fig. 1). cocci-call was benchmarked on an HPC system using 28–64 CPU cores and 128–512 GB of RAM. For single-sample runs, the pipeline consumed an average of 1.07 ± 0.32 CPU hours per sample, depending on genome size and region filtering. The pipeline supports parallel execution and is fully containerized for portability and consistency across platforms.

### Benchmarking data sets

We evaluated the impact of genomic region selection on variant identification in *Coccidioides* using two complementary benchmarking strategies: a simulation-based strategy and an empirical strategy. For the simulation-based strategy, we used two reference genomes—*C*. *immitis* (GCA_004115165.2) and *C. posadasii* (GCA_000151335.1)—to generate multiple independent simulated data sets using ART (17). For each species, we simulated 10 replicate short-read data sets with a median coverage of 100× and sequencing error modeled after the Illumina HiSeq 2500 (HS25) platform. These simulations allowed us to define true variant positions and systematically assess variant calling performance. Notably, the genomes used for simulation differed from those used as references in the *cocci-call* pipeline (GCF_000149335.2 for *C. immitis* and GCA_018416015.2 for *C. posadasii*), enabling evaluation of mapping bias and reference choice effects.

For the empirical strategy, we sequenced the *C. posadasii *NR-166 strain (Δcts2/Δard1/Δcts3), a widely used laboratory strain with well-characterized genetic variation. DNA was extracted using the QIAamp DNA Mini Kit, libraries were prepared with the NEBNext Ultra II FS DNA Library Prep Kit, and sequencing was performed on a NovaSeq X Series 10B (150 × 150 bp) at the University of Utah High-Throughput Genomics Shared Resource.

To identify repetitive elements, we used RepeatMasker v4.1.9 (http://www.repeatmasker.org/) with default settings and NUCmer v3.1 (18), aligning each genome to itself. Repetitive regions were inferred from areas where self-alignments were imperfect, reflecting regions of sequence redundancy. NUCmer was run with the parameters: “--maxmatch and—mincluster 100.” Delta-filter was applied with the “-g” option to retain the best alignments, and show-snps was used with the options “-C, -I, -r, and -T” to extract variant positions.

### Measuring performance in alternative benchmarking regions

To assess the impact of genomic region selection on variant calling accuracy in *Coccidioides*, we compared variant identification across three genomic contexts: (i) the whole genome, (ii) regions with repetitive elements masked, and (iii) gene-only regions. In addition to cocci-call, we also evaluated variant calling performance using the MycoSNP pipeline (18). We then benchmarked small variant calls with vcfdist (19) against the “truth” VCF files described above. Genomic analyses in well-characterized species often rely on standardized benchmarking regions that typically exclude repetitive regions (9). To determine which regions of the *Coccidioides* genome should be considered or excluded for variant calling, we used RepeatMasker (https://www.repeatmasker.org/) and NUCmer (20) to identify repetitive elements. We then assessed variant-calling performance across alternative genomic regions, including the full genome, gene regions, and regions masked or unmasked by RepeatMasker and/or NUCmer. For each region, we calculated the number of true positives, false positives, true negatives, and false negatives to evaluate model performance using precision, recall, and the F1 score. Precision was defined as the proportion of true positives among all predicted positives, while recall represented the proportion of true positives among all actual positives. The F1 score, calculated as the harmonic mean of precision and recall, provided a single metric to balance false positives and false negatives. This approach simplifies evaluation by combining precision and recall into a single performance metric. Additionally, we calculated the Jaccard index as the number of shared SNPs between pipelines divided by the total number of unique SNPs to measure concordance in SNP detection across regions. All figures were generated in R using the ggplot2 package or in Python using the pyplot and seaborn packages (21–23).

## RESULTS

### Variant identification performance prior to masking

**Fig 2:**
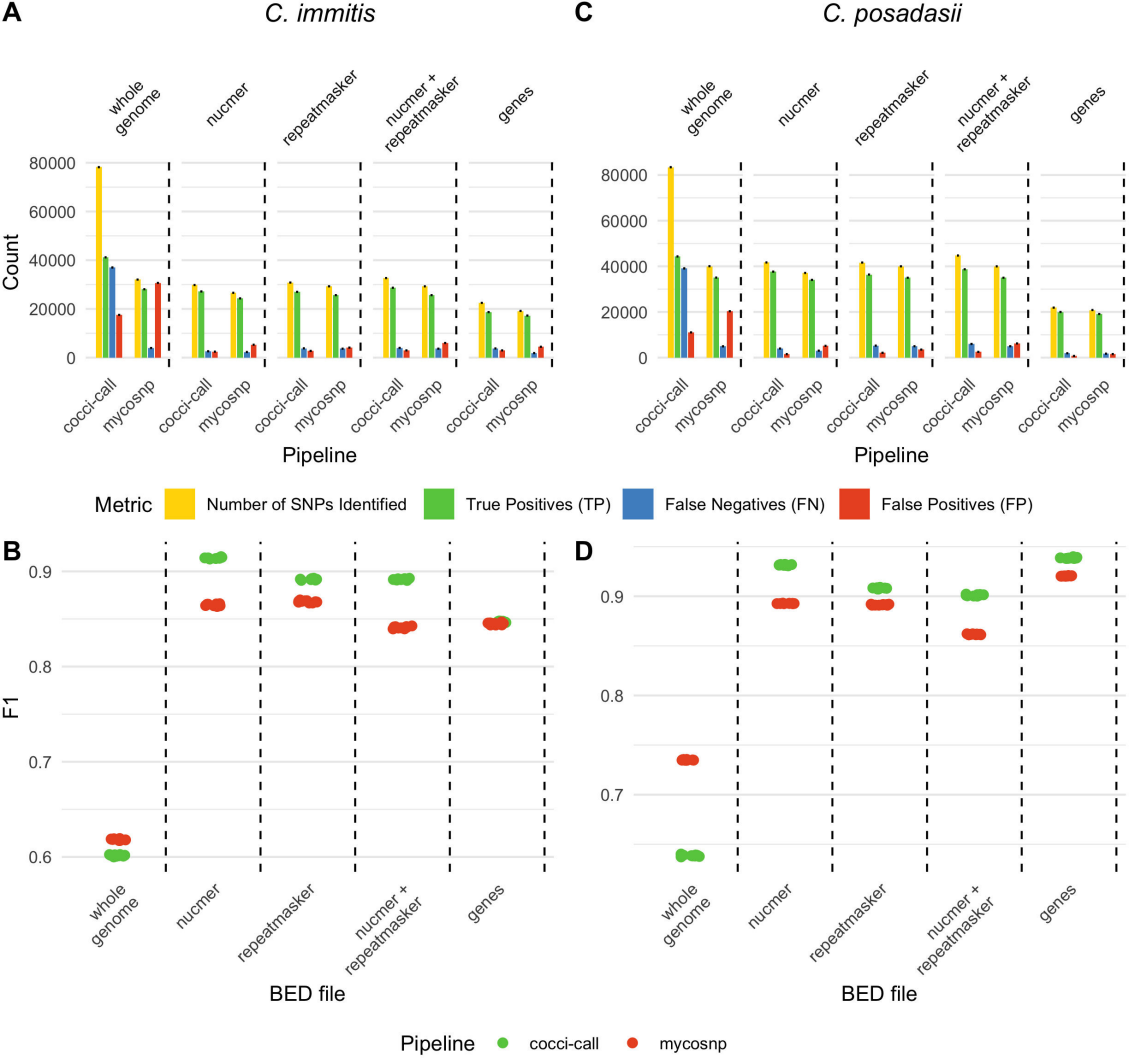
Magnitude of *Coccidioides* variants identified and performance across benchmarking regions for *in silico* sequence data. Bars show the median count across replicates, with error bars indicating standard error. Total SNPs (yellow), true positives (TP, green), false negatives (FN, blue), and false positives (FP, red) identified by the cocci-call and MycoSNP pipelines for (**A**) *C. immitis* and (**B**) *C. posadasii*. Vertical lines delimit different benchmarking regions: the whole genome, NUCmer-masked, RepeatMasker-masked, combined NUCmer and RepeatMasker-masked, and gene-only regions. Panels **C** and **D** show the F1 scores of the cocci-call (green) and MycoSNP (red) pipelines across the same benchmarking regions.

To assess where variants should be confidently called, we analyzed SNP detection across *in silico* whole-genome data sets for *C. immitis* and *C. posadasii* using two independent variant-calling pipelines, cocci-call and MycoSNP, a previously published pipeline (18). In the *C. immitis* data sets, cocci-call identified a median of 78,182 SNPs (IQR: 78,104-78,334), while MycoSNP detected a median of 38,065 SNPs (IQR: 38,053-32,111) across 10 simulated data sets (Fig. 2A). Among these, 32,067 of the SNPs were consistently detected by both pipelines, highlighting a core overlap that underscores shared detection capabilities. This overlap constitutes approximately 41% of the SNPs identified by cocci-call and 84% of those detected by MycoSNP. The Jaccard index of 0.34 for *C. immitis* indicates a moderate level of concordance between cocci-call and MycoSNP.

Both cocci-call and MycoSNP achieved similar F1 scores on the unmasked genomes, pointing to comparable accuracy (Fig. 2C). Yet, a detailed examination reveals contrasting strengths: cocci-call demonstrated higher sensitivity (70.1%, IQR: 70.0%–70.1%), capturing a larger proportion of true positive variants, whereas MycoSNP showed significantly higher precision (87.5%, IQR: 87.5%–87.6%), indicating fewer false positives (Fig. 4B; Fig. S1A–B). The resulting F1 scores were 60.1% (IQR: 60.0%–60.2%) for cocci-call and 61.8% (IQR: 61.7%–61.8%) for MycoSNP (Fig. 2B).

**Fig 3:**
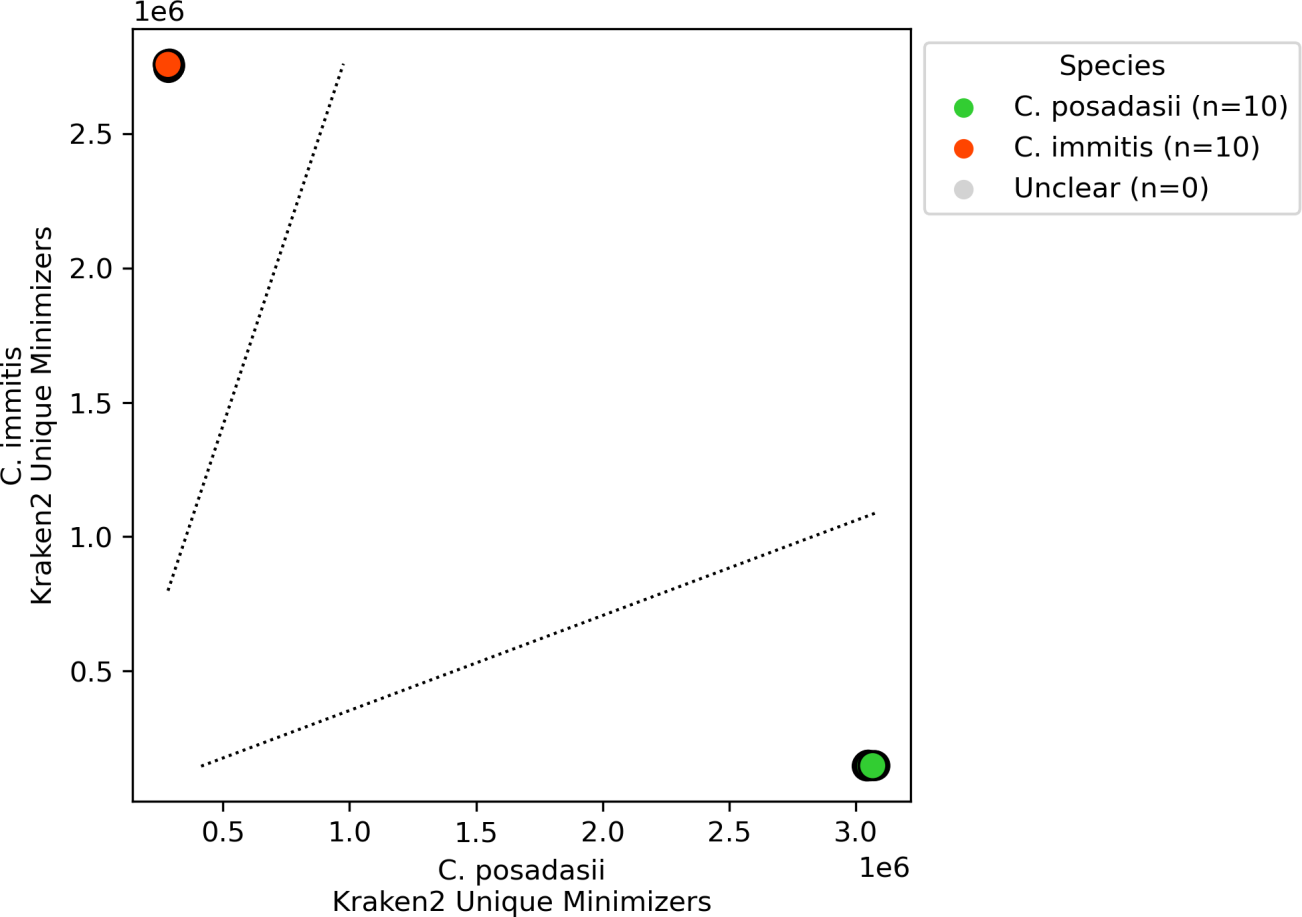
Accuracy of *Coccidioides* species assignment by cocci-call for *in silico* sequence data. Points indicate unique sequence data generated *in silico* from reference genomes of both *C. immitis* and *C. posadasii* (indicated by point color). Points are located along the *x*-axis by the number of k-mers that uniquely map to the *C. posadasii* reference genome and along the *y*-axis by the number of k-mers that uniquely map to the *C. immitis* reference genome. The dashed line denotes the species assignment threshold, demonstrating cocci-call’s ability to distinguish between *C. posadasii* and *C. immitis*.

**Fig 4:**
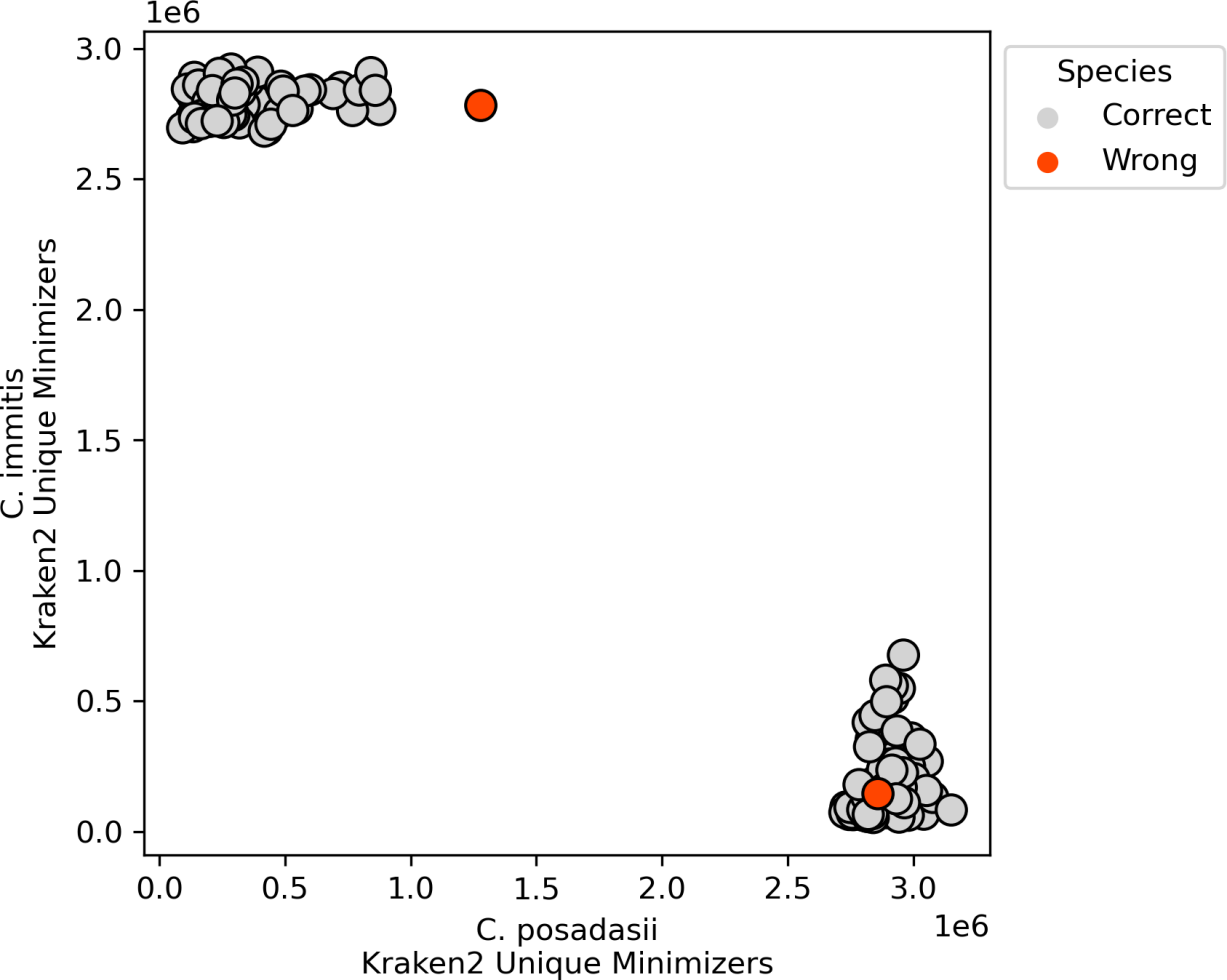
Accuracy of *Coccidioides* species assignment by cocci-call for previously published *Coccidioides* sequence data. Gray circles represent correctly assigned samples, with clusters corresponding to *C. posadasii* and *C. immitis* based on Kraken2 minimizer values along the horizontal and vertical axes, respectively. The orange circles indicate misclassified samples.

In *C. posadasii*, the trends were similar. Here, cocci-call identified a median of 83,400 SNPs, while MycoSNP detected 40,018 SNPs (Fig. 2B). Of the total SNPs identified, 40,163 were detected by both cocci-call and MycoSNP. This accounts for 38.9% of the SNPs identified by cocci-call and 79.7% of those detected by MycoSNP, with a Jaccard index of 0.36 for *C. posadasii*, indicating a moderate level of agreement between the two pipelines for this species.

Further analysis of *C. posadasii* variant calling revealed differences in tool performance. While MycoSNP attained a higher F1 score (73.4%, IQR: 73.4%–73.5%) compared with cocci-call’s 63.8% (IQR: 63.8%–63.8%), cocci-call demonstrated a notably higher sensitivity, detecting 79.9% (IQR: 79.9%–80.0%) of true variants versus MycoSNP’s 63.3% (IQR: 63.2%–63.3%) (Fig. 2; S1C). However, MycoSNP outperformed cocci-call in precision, achieving 87.6% (IQR: 87.5%–87.6%) compared with cocci-call’s 53.1% (IQR: 53.0%–53.1%) (Fig. S1D).

### Choice of benchmarking region impacts performance of *Coccidioides* variant identification

We compared repetitive genomic regions identified by two commonly used tools for repeat identification. Overall, both NUCmer and RepeatMasker identify similar repetitive regions in both species (Fig. S2). However, certain contigs (e.g., *C. immitis*: NW_004504306.1; *C. posadasii*: CP075073.1 and CP075074.1) showed significantly less overlap in repetitive regions identified by the two tools, with RepeatMasker identifying most of the repetitive regions in these cases. This discrepancy highlights variability in repetitive region detection across specific genomic regions and underscores the importance of using multiple tools to achieve comprehensive coverage.

The selection of benchmarking regions—i.e., the specific portions of the genome where performance is evaluated—had a substantial impact on the results of variant calling. Analyses focused on gene-only regions or those excluding repetitive sequences consistently yielded higher F1 scores compared with genome-wide assessments. This pattern underscores a key tradeoff: while genome-wide approaches identify a greater number of total SNPs, they are more susceptible to false positives introduced by repetitive elements. In contrast, restricting analysis to high-confidence regions—either by masking repeats or targeting genes—enhances accuracy by reducing false variant calls, though at the cost of detecting fewer SNPs overall (Fig. 2).

This trade-off is clearly illustrated in our benchmarking of cocci-call. For *C. immitis*, genome-wide analysis identified 90,570 SNPs and 41,181 true positives (TPs), with a median sensitivity of 70.1% and precision of 52.7%. When repetitive regions were masked using RepeatMasker alone, SNPs dropped to 34,582 and true positives to 27,018, with improved sensitivity (90.8%) and precision (87.6%). Masking with Nucmer alone yielded 32,935 SNPs and 27,164 TPs, with even better performance (91.7% sensitivity, 91.1% precision). A gene-only approach detected 23,929 SNPs and 18,716 TPs, with sensitivity and precision reaching 86.2% and 83.2%, respectively.

A similar trend was observed for *C. posadasii*. Genome-wide, cocci-call identified 95,010 SNPs and 44,322 TPs (80.0% sensitivity, 53.1% precision). Masking with RepeatMasker reduced SNPs to 36,378 and yielded 36,378 TPs (94.5% sensitivity, 87.4% precision), while Nucmer masking identified 37,684 TPs from 37,684 SNPs (96.1% sensitivity, 90.4% precision). In gene-only regions, cocci-call reported 24,598 SNPs and 19,998 TPs, achieving 96.5% sensitivity and 91.3% precision.

For *C. immitis*, cocci-call’s F1 score increased from 60.2% (IQR: 60.1%–60.2%) to 84.7% (IQR: 84.6%–84.8%) when analysis was limited to gene-only regions (Fig. 2B). Similarly, MycoSNP’s F1 score rose from 61.9% (IQR: 61.8%–61.9%) to 84.5% (IQR: 84.4%–84.6%) under the same conditions (Fig. 2B). Masking repetitive regions using NUCmer further elevated performance, with cocci-call achieving an F1 score of 91.4% (IQR: 91.4%–91.5%) and MycoSNP reaching 86.5% (IQR: 86.4%–86.5%) (Fig. 3B). When additional filtering was applied using RepeatMasker, alone or combined with NUCmer, similar trends emerged (Fig. 2B).

cocci-call and MycoSNP were largely consistent in SNP identification. In gene-only regions, for example, 71% of total unique SNPs overlapped, encompassing 80% of cocci-call and 87% of MycoSNP SNPs, resulting in a Jaccard index of 0.71. RepeatMasker-masked regions showed a stronger concordance, with 78% of total unique SNPs overlapping, covering 93% of cocci-call and 83% of MycoSNP SNPs, yielding a Jaccard index of 0.78. NUCmer-masked regions exhibited a similarly high overlap, with 79% of unique SNPs shared, covering 88% of cocci-call and 89% of MycoSNP SNPs, reflected in a Jaccard index of 0.79. The masked union, combining RepeatMasker and NUCmer regions, demonstrated a SNP overlap of 74%, representing 88% of cocci-call and 83% of MycoSNP SNPs, with a Jaccard index of 0.74.

Similar to *C. immitis*, restricting the analysis to gene-only regions in *C. posadasii* resulted in a significant improvement, with cocci-call’s F1 score rising markedly from 63.9% (IQR: 63.8%–63.9%) to 93.8% (IQR: 93.8%–93.9%) (Fig. 2D). MycoSNP’s F1 increased from 73.5% (IQR: 73.5%–73.5%) to 92.0% (IQR: 92.0%–92.1%) (Fig. 2D). Similarly to *C. immitis*, masking repetitive regions using NUCmer produced considerable gains, with cocci-call achieving an F1 score of 93.1% (IQR: 93.1%–93.2%) and MycoSNP reaching 89.3% (IQR: 89.3%–89.3%). When further filtering was applied using RepeatMasker, cocci-call maintained high accuracy with an F1 score of 90.8% (IQR: 90.8%–90.8%), while MycoSNP scored 89.1% (IQR: 89.1%–89.2%) (Fig. 3D). Combining NUCmer and RepeatMasker also proved beneficial, with cocci-call achieving 90.1% (IQR: 90.1%–90.2%) and MycoSNP reaching 86.2% (IQR: 86.1%–86.2%) (Fig. 2D).

In gene-only regions, cocci-call and MycoSNP demonstrated a high level of concordance, with approximately 84% of cocci-call SNPs and 90% of MycoSNP SNPs overlapping, leading to a Jaccard index of 0.84. RepeatMasker-masked regions showed similarly robust agreement, with 75% of total unique SNPs overlapping, representing 92% of cocci-call and 80% of MycoSNP SNPs, and a Jaccard index of 0.75. NUCmer-masked regions exhibited an even higher overlap, with 79% of total unique SNPs shared, covering 87% of cocci-call and 89% of MycoSNP SNPs, corresponding to a Jaccard index of 0.79. The intersection of RepeatMasker and NUCmer-masked regions reflected high concordance, with 71% of unique SNPs overlapping, accounting for 86% of cocci-call and 80% of MycoSNP SNPs, yielding a Jaccard index of 0.71.

### Variant identification performance in empirically generated sequence data

**Fig 5:**
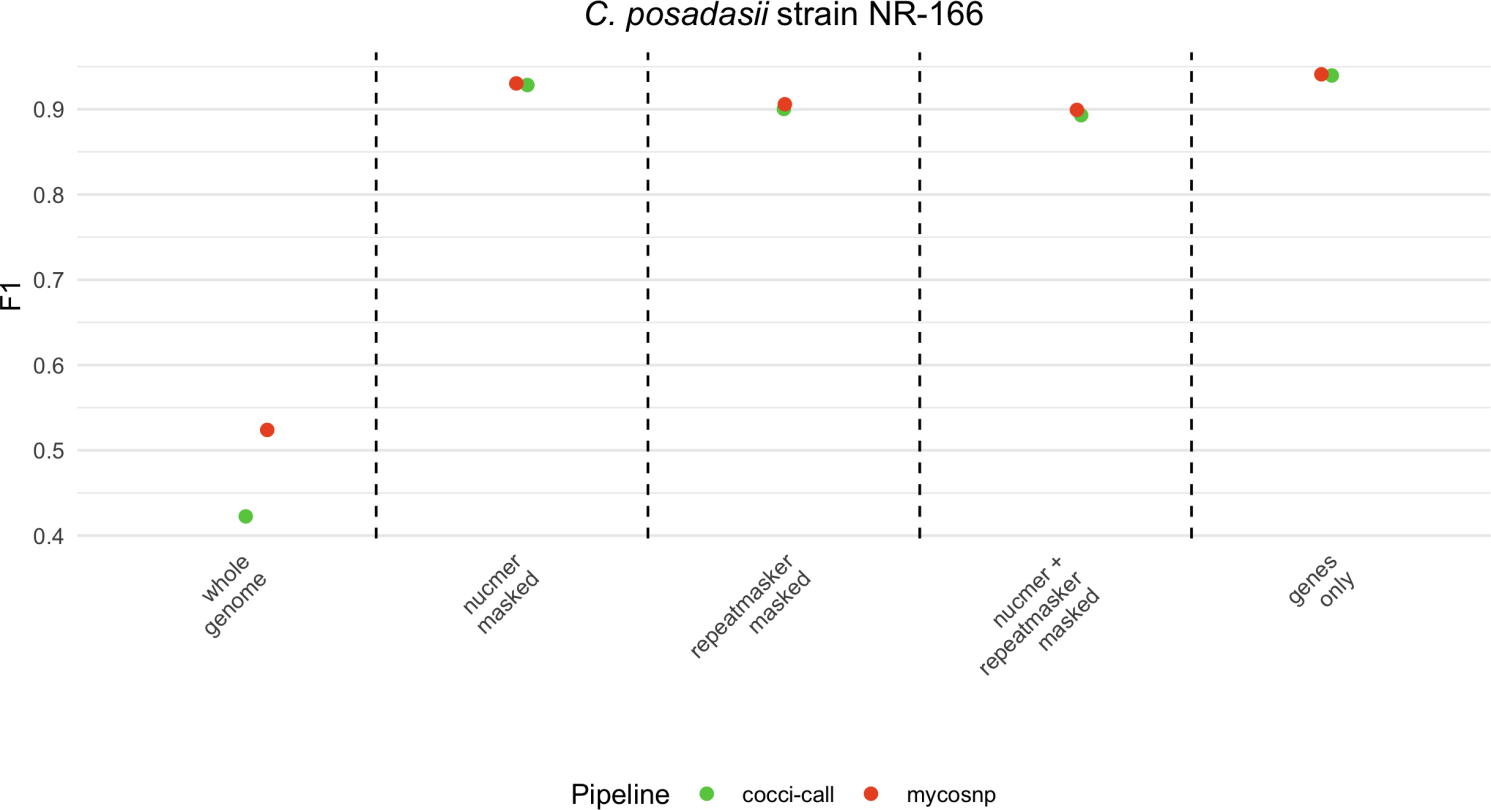
Performance of variant identification across benchmarking regions for empirically generated sequence data. Comparison of F1 scores for variant detection by cocci-call (green) and MycoSNP (red) across different genomic regions in *C. posadasii* strain NR-166. Vertical lines delimit different benchmarking regions: the whole genome, NUCmer-masked, RepeatMasker-masked, combined NUCmer and RepeatMasker-masked, and gene-only regions.

To identify the most reliable regions for variant calling in *Coccidioides*, we assessed genome-wide performance using sequence data from *C. posadasii* strain NR-166. Sequencing of this strain yielded high-quality data, with an average coverage of 226× (SD: 69×) and a median coverage of 250×. Approximately 95% of the genome was covered at ≥10× , and 99.6% of reads mapped to the reference genome with 93.1% properly paired and no PCR duplicates detected. These results confirm high sequencing depth and alignment quality, ensuring confidence in downstream variant analysis. Across the entire genome, F1 scores were 63.9% (IQR: 63.8%–63.9%) for cocci-call and 73.5% (IQR: 73.5%–73.5%) for MycoSNP. Focusing on gene-only regions, cocci-call’s F1 score improved significantly to 93.8% (IQR: 93.8%–93.9%), while MycoSNP reached 92.0% (IQR: 92.0%–92.1%) (Fig. 4). Masking repetitive regions with NUCmer further enhanced cocci-call’s F1 to 93.1% (IQR: 93.1%–93.2%) and MycoSNP’s to 89.3% (IQR: 89.3%–89.3%) (Fig. 5). Even when using RepeatMasker or a combined approach with both NUCmer and RepeatMasker, cocci-call continued to perform better than MycoSNP, although both pipelines experienced slight F1 decreases in masked repetitive regions (Fig. 5).

### Species identification performance

cocci-call assigned *Coccidioides* species in 20 *in silico* read sets (10 from *C. immitis* and 10 from *C. posadasii*) with 100% accuracy (Fig. 3). We additionally tested the performance of cocci-call in identifying species in publicly available sequence data from previous studies, using the species assigned by the submitting study as the gold standard. Among these empirically collected isolates, cocci-call assigned species with 98.86% (173 of 175) accuracy (Fig. 4). A single sample, which was labeled unknown by the submitting study, was not definitively assigned to a species, but had a log-transformed *immitis*/*posadasii* Kraken2 minimizers ratio of 1.12, suggesting it was likely *C. immitis* or a *C. immitis* strain with introgressed regions (Fig. 4). In another sample, cocci-call assigned *C. posadasii,* though the submitting study assigned the sample *C. immitis;* this was potentially an instance of misassignment in the original study (Fig. 4).

## DISCUSSION

Accurate variant identification is crucial for understanding the genomic diversity of *Coccidioides* and to harness variation for genomic epidemiology and phylogenetics (e.g., 3, 5, 8). Here, we developed a novel bioinformatic pipeline for *Coccidioides* variant identification and species assignment and benchmarked our pipeline in different genomic regions. We found that (i) accuracy of variant calls improved significantly outside of repetitive regions and inside genes as compared with the across the whole genome, (ii) tradeoffs in total variation identified with different approaches may impact bioinformatic choice, and (iii) *Coccidioides* species can be identified with near-perfect accuracy when compared with species labels from submitting laboratory.

Our results reveal a critical tradeoff between sensitivity and precision in variant calling across different genomic regions. Whole-genome analyses, while capturing a broader range of variants, are more susceptible to sequencing artifacts, which can compromise accuracy (24). In contrast, restricting analyses to gene-rich regions or masking repetitive elements significantly improves precision, as evidenced by the substantial increase in F1 scores, from 63.9% to 93.8% in *C. posadasii* and from 60.2% to 84.7% in *C. immitis*. These results highlight the importance of carefully selecting genomic regions to optimize variant calling, particularly in fungal species like *Coccidioides*, where balancing sensitivity and specificity is crucial.

To achieve these improvements, specific strategies should be integrated into variant calling pipelines. Masking repetitive regions using tools like RepeatMasker and NUCmer is a foundational step, as it reduces noise and enhances the reliability of variant detection. Additionally, focusing on gene-rich regions ensures that biologically meaningful variants are prioritized, further refining the balance between sensitivity and specificity. While both approaches improve accuracy, masking repetitive elements alone has the added benefit of preserving a greater number of SNPs, making it a particularly effective strategy for *Coccidioides* variant calling. Together, these methods provide a robust framework for optimizing pipelines and ensuring high-quality results.

Beyond improving variant identification, species classification accuracy is critical for genomic epidemiology. cocci-call incorporates Kraken2-based taxonomic filtering (12), which provides a powerful and efficient alternative to traditional phylogenetic methods, particularly for species classification in complex metagenomic samples. By leveraging k-mer matching and curated reference databases, this approach not only enhances species assignment accuracy but also streamlines the identification of *Coccidioides* while minimizing false positives. This makes it especially valuable for large-scale genomic epidemiology studies, where accurate species inference is crucial for tracking pathogen spread and informing public health interventions. Our study demonstrates that cocci-call achieves a species classification accuracy of 98.86% on publicly available data sets, further validating its utility in fungal genomic research.

Our results show that cocci-call consistently detects more SNPs than MycoSNP (18), particularly in whole-genome comparisons. This difference likely reflects cocci-call’s per-sample variant calling strategy, which increases sensitivity to low-frequency variants often missed by joint-calling approaches. While MycoSNP’s joint calling can enhance precision in population-scale studies, cocci-call offers greater flexibility for diverse research and clinical contexts, particularly those involving limited or individual-level samples. This tradeoff highlights how pipeline design can shape variant discovery and should be considered when tailoring workflows to specific study goals. Another advantage of cocci-call over MycoSNP when processing *Coccidioides* samples is the early species identification step, which results in subsequent analyses (alignment and variant calling) to be performed using the correct species reference. MycoSNP, on the other end, would need to be run twice, i.e., once for *C. immitis* and once for *C. posadasii*, resulting in longer computation time and resource expenditure.

The cocci-call pipeline offers a powerful framework for both clinical diagnostics and genomic epidemiology of *Coccidioides* spp. Its accurate species identification and high-resolution variant detection enable precise infection tracking, particularly in cases involving travel or emerging endemicity. This capability is critical for guiding treatment and informing public health responses. Beyond individual cases, cocci-call promotes standardization across studies, facilitating consistent variant calls and enabling large-scale comparative analyses. By combining sensitivity, precision, and portability, cocci-call stands out as a key tool for advancing fungal genomics and strengthening surveillance of this medically important pathogen.

What sets cocci-call apart is its integration of features not available in other tools. First, it uniquely benchmarks variant calling accuracy across gene-rich, repetitive, and whole-genome regions in *Coccidioides*, allowing users to evaluate performance in distinct genomic contexts. Second, it incorporates species identification directly from sequencing reads using Kraken2, which is especially valuable in clinical and metagenomic applications. Third, it is implemented in Nextflow with Docker containers, ensuring reproducibility, portability, and scalability on high-performance computing systems. While cocci-call employs well-established tools internally (e.g., BWA, GATK), its innovation lies in its fungal-specific design, modular architecture, and benchmarking framework. By combining sensitivity, precision, and usability, cocci-call is positioned as a key resource for advancing fungal genomics and strengthening surveillance of this medically important pathogen.

Long-read sequencing technologies, such as PacBio and Nanopore, could resolve complex structural variations. Expanding variant detection beyond SNPs to include insertions, deletions, and copy number variations will provide a more comprehensive understanding of *Coccidioides* genome evolution. In addition, future work should explore benchmarking alternative alignment and variant calling tools, such as FreeBayes (25), Bowtie2 (26), or bwa-mem2 (27), to evaluate whether they offer advantages in specific genomic contexts. This will help ensure that cocci-call remains flexible and robust across a range of sequencing platforms and analysis goals.

The complexity of *Coccidioides* genomes and the tradeoffs between variant identification specificity and sensitivity may mean that different approaches are best suited for different downstream analyses. To address this, our bioinformatic pipeline allows users to choose benchmarking region. More generally, we find that the use of benchmarked variant identification tools may improve the reproducibility and comparability of *Coccidioides* genomic epidemiology studies.

## Data Availability

Raw sequencing data and the variant identification pipeline (cocci-call), along with all analysis scripts, are publicly available under BioSample SAMN45887705 and at https://github.com/ksw9/cocci-call.

## SUPPLEMENTAL MATERIAL

Figure S1 and S2 spectrum.01232-25-s0001.pdf Fig. S1: Comparison of precision and recall for *Coccidioides* variant identification. Fig. S2: Repetitive genomic regions identified by different tools.

ASM does not own the copyrights to Supplemental Material that may be linked to, or accessed through, an article. The authors have granted ASM a non-exclusive, world-wide license to publish the Supplemental Material files. Please contact the corresponding author directly for reuse.

## References

[1] Fisher MC, Gurr SJ, Cuomo CA, Blehert DS, Jin H, Stukenbrock EH, Stajich JE, Kahmann R, Boone C, Denning DW, Gow NAR, Klein BS, Kronstad JW, Sheppard DC, Taylor JW, Wright GD, Heitman J, Casadevall A, Cowen LE. 2020. Threats posed by the fungal kingdom to humans, wildlife, and agriculture. mBio 11:e00449-20. doi:10.1128/mBio.00449-20 32371596 PMC7403777

[2] WHO. 2022. WHO fungal priority pathogens list to guide research, development and public health action. World Health Organization. https://www.who.int/publications/i/item/9789240060241.

[3] Oltean HN, Etienne KA, Roe CC, Gade L, McCotter OZ, Engelthaler DM, Litvintseva AP. 2019. Utility of whole-genome sequencing to ascertain locally acquired cases of coccidioidomycosis, Washington, USA. Emerg Infect Dis 25:501–506. doi:10.3201/eid2503.181155 30789132 PMC6390764

[4] Barker BM, Litvintseva AP, Riquelme M, Vargas-Gastélum L. 2019. Coccidioides ecology and genomics. Med Mycol Open Access 57:S21–S29. doi:10.1093/mmy/myy051 PMC6347077 30690605

[5] Engelthaler DM, Roe CC, Hepp CM, Teixeira M, Driebe EM, Schupp JM, Gade L, Waddell V, Komatsu K, Arathoon E, Logemann H, Thompson GR, Chiller T, Barker B, Keim P, Litvintseva AP. 2016. Local population structure and patterns of western hemisphere dispersal for Coccidioides spp., the fungal cause of valley fever. mBio 7:e00550-16. doi:10.1128/mBio.00550-16 27118594 PMC4850269

[6] McCotter OZ, Benedict K, Engelthaler DM, Komatsu K, Lucas KD, Mohle-Boetani JC, Oltean H, Vugia D, Chiller TM, Sondermeyer Cooksey GL, Nguyen A, Roe CC, Wheeler C, Sunenshine R. 2019. Update on the epidemiology of coccidioidomycosis in the United States. Med Mycol Open Access 57:S30–S40. doi:10.1093/mmy/myy095 PMC6823633 30690599

[7] de Melo Teixeira M, Stajich JE, Sahl JW, Thompson GR III, Brem RB, Dubin CA, Blackmon AV, Mead HL, Keim P, Barker BM. 2022. A chromosomal-level reference genome of the widely utilized Coccidioides posadasii laboratory strain “Silveira”. G3 (Bethesda) 12. doi:10.1093/g3journal/jkac031 PMC8982387 35137016

[8] Neafsey DE, Barker BM, Sharpton TJ, Stajich JE, Park DJ, Whiston E, Hung C-Y, McMahan C, White J, Sykes S, et al.. 2010. Population genomic sequencing of Coccidioides fungi reveals recent hybridization and transposon control. Genome Res 20:938–946. doi:10.1101/gr.103911.109 20516208 PMC2892095

[9] Krusche P, Trigg L, Boutros PC, Mason CE, De La Vega FM, Moore BL, Gonzalez-Porta M, Eberle MA, Tezak Z, Lababidi S, Truty R, Asimenos G, Funke B, Fleharty M, Chapman BA, Salit M, Zook JM, Global Alliance for Genomics and Health Benchmarking Team. 2019. Best practices for benchmarking germline small-variant calls in human genomes. Nat Biotechnol 37:555–560. doi:10.1038/s41587-019-0054-x 30858580 PMC6699627

[10] Li H, Bloom JM, Farjoun Y, Fleharty M, Gauthier L, Neale B, MacArthur D. 2018. A synthetic-diploid benchmark for accurate variant-calling evaluation. Nat Methods 15:595–597. doi:10.1038/s41592-018-0054-7 30013044 PMC6341484

[11] Olson ND, Wagner J, Dwarshuis N, Miga KH, Sedlazeck FJ, Salit M, Zook JM. 2023. Variant calling and benchmarking in an era of complete human genome sequences. Nat Rev Genet 24:464–483. doi:10.1038/s41576-023-00590-0 37059810

[12] Wood DE, Lu J, Langmead B. 2019. Improved metagenomic analysis with Kraken 2. Genome Biol 20:257. doi:10.1186/s13059-019-1891-0 31779668 PMC6883579

[13] Li H, Durbin R. 2009. Fast and accurate short read alignment with Burrows-Wheeler transform. Bioinformatics 25:1754–1760. doi:10.1093/bioinformatics/btp324 19451168 PMC2705234

[14] McKenna A, Hanna M, Banks E, Sivachenko A, Cibulskis K, Kernytsky A, Garimella K, Altshuler D, Gabriel S, Daly M, DePristo MA. 2010. The genome analysis toolkit: a MapReduce framework for analyzing next-generation DNA sequencing data. Genome Res 20:1297–1303. doi:10.1101/gr.107524.110 20644199 PMC2928508

[15] Cingolani P, Platts A, Wang LL, Coon M, Nguyen T, Wang L, Land SJ, Lu X, Ruden DM. 2012. A program for annotating and predicting the effects of single nucleotide polymorphisms, SnpEff: SNPs in the genome of Drosophila melanogaster strain w1118; iso-2; iso-3. Fly (Austin) 6:80–92. doi:10.4161/fly.19695 22728672 PMC3679285

[16] Li H. 2011. A statistical framework for SNP calling, mutation discovery, association mapping and population genetical parameter estimation from sequencing data. Bioinformatics 27:2987–2993. doi:10.1093/bioinformatics/btr509 21903627 PMC3198575

[17] Huang W, Li L, Myers JR, Marth GT. 2012. ART: a next-generation sequencing read simulator. Bioinformatics 28:593–594. doi:10.1093/bioinformatics/btr708 22199392 PMC3278762

[18] Bagal UR, Phan J, Welsh RM, Misas E, Wagner D, Gade L, Litvintseva AP, Cuomo CA, Chow NA. 2022. MycoSNP: a portable workflow for performing whole-genome sequencing analysis of Candida auris. Methods Mol Biol 2517:215–228. doi:10.1007/978-1-0716-2417-3_17 35674957

[19] Dunn T, Narayanasamy S. 2023. Vcfdist: accurately benchmarking phased small variant calls in human genomes. Nat Commun 14:8149. doi:10.1038/s41467-023-43876-x 38071244 PMC10710436

[20] Kurtz S, Phillippy A, Delcher AL, Smoot M, Shumway M, Antonescu C, Salzberg SL. 2004. Versatile and open software for comparing large genomes. Genome Biol 5:R12. doi:10.1186/gb-2004-5-2-r12 14759262 PMC395750

[21] Wickham H. 2009. Ggplot2: elegant graphics for data analysis. Springer, New York.

[22] Hunter JD. 2007. Matplotlib: a 2D graphics environment. Comput Sci Eng 9:90–95. doi:10.1109/MCSE.2007.55

[23] Waskom M. 2021. Seaborn: statistical data visualization. JOSS 6:3021. doi:10.21105/joss.03021

[24] DePristo MA, Banks E, Poplin R, Garimella KV, Maguire JR, Hartl C, Philippakis AA, del Angel G, Rivas MA, Hanna M, McKenna A, Fennell TJ, Kernytsky AM, Sivachenko AY, Cibulskis K, Gabriel SB, Altshuler D, Daly MJ. 2011. A framework for variation discovery and genotyping using next-generation DNA sequencing data. Nat Genet 43:491–498. doi:10.1038/ng.806 21478889 PMC3083463

[25] Garrison E, Marth G. 2012. Haplotype-based variant detection from short-read sequencing. arXiv. doi:10.48550/arXiv.1207.3907

[26] Langmead B, Salzberg SL. 2012. Fast gapped-read alignment with Bowtie 2. Nat Methods 9:357–359. doi:10.1038/nmeth.1923 22388286 PMC3322381

[27] Vasimuddin M, Misra S, Li H, Aluru S. 2019. Efficient architecture-aware acceleration of BWA-MEM for multicore systems. 2019 IEEE International Parallel and Distributed Processing Symposium (IPDPS); Rio de Janeiro, Brazil: , Vol. 2019, p 314–324. doi:10.1109/IPDPS.2019.00041

