# Supplementary material for: The impact of bioinformatic choices on Coccidioides variant identification accuracy
Source: Microbiol Spectr. 2025 Aug 15;13(10):e01232-25. doi: 10.1128/spectrum.01232-25 (PMC12502711; doi:10.1128/spectrum.01232-25)
Supplement: Figure S1 and S2 — Fig. S1: Comparison of precision and recall for Coccidioides variant identification. Fig. S2: Repetitive genomic regions identified by different tools. [file spectrum.01232-25-s0001.pdf]

SUPPORTING INFORMATION

The impact of bioinformatic choices on *Coccidioides* variant identification accuracy

Marco Marchetti[1], Emanuel M. Fonseca[2], Kimberly E. Hanson[3,4], Bridget Barker[5], Katharine S. Walter[6]

[1] Eccles Institute of Human Genetics, University of Utah, Salt Lake City, UT, 84112, USA
[2] Elfa Analytics, Ponte Nova, Minas Gerais, BR, 35430-223
[3] Department of Medicine, Division of Infectious Diseases, University of Utah School of Medicine, Salt Lake City, Utah, USA
[4] Department of Pathology, Division of Clinical Microbiology, University of Utah and ARUP Laboratories, Salt Lake City, Utah, USA
[5] Department of Biological Sciences, Northern Arizona University, Flagstaff, AZ, USA
[6] Division of Epidemiology, University of Utah, Salt Lake City, UT 84105, USA

Correspondence: Katharine S. Walter, katharine.walter@hsc.utah.edu

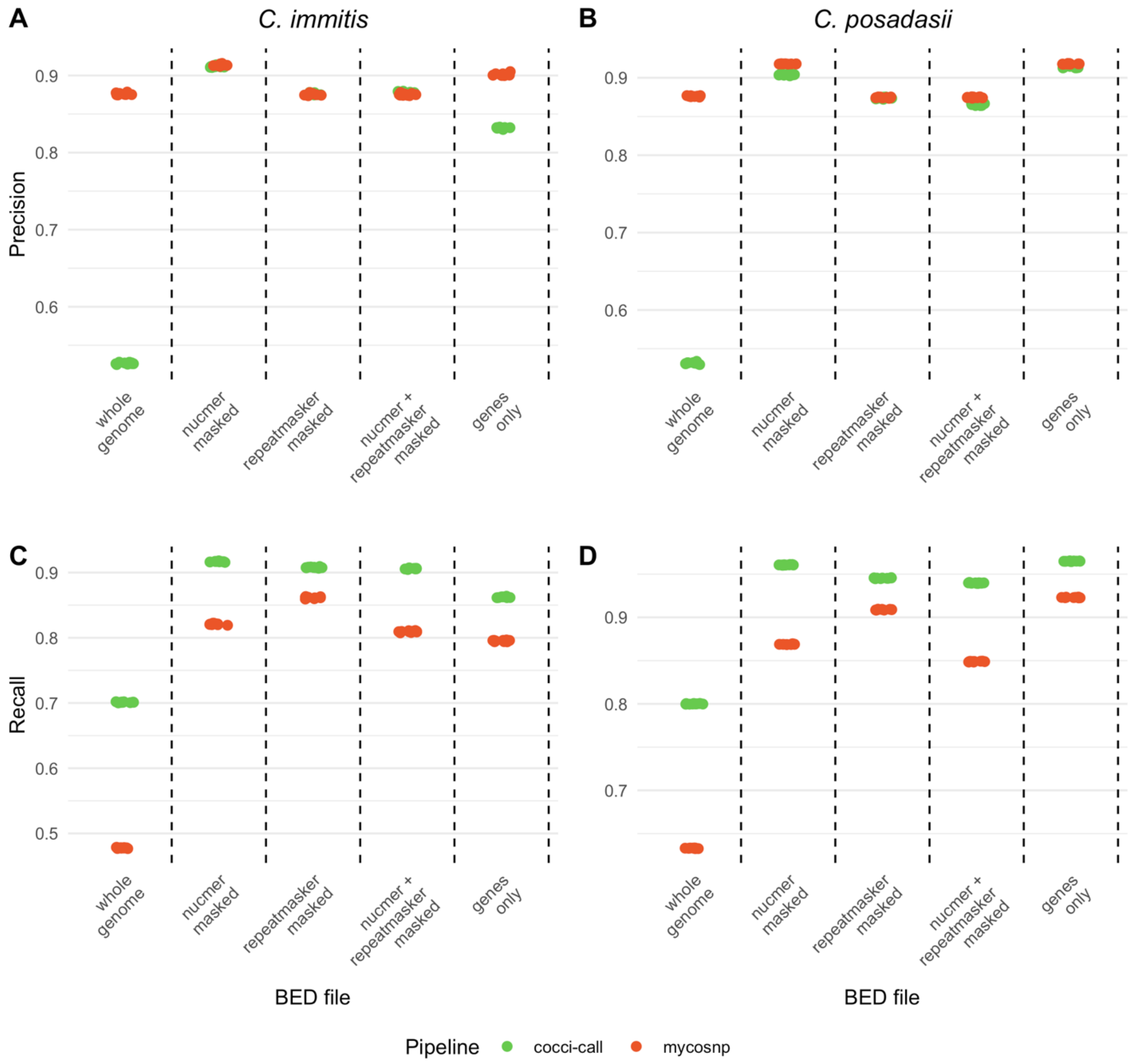

Fig. S1. Comparison of precision and recall for *Coccidioides* variant identification. (A,B) Precision and (C,D) recall of variants identified by cocci-call (green) and MycoSNP (orange). Horizontal facets indicate benchmarking region: the whole genome, NUCmer-masked, RepeatMasker-masked, combined NUCmer and RepeatMasker-masked, and gene-only regions.

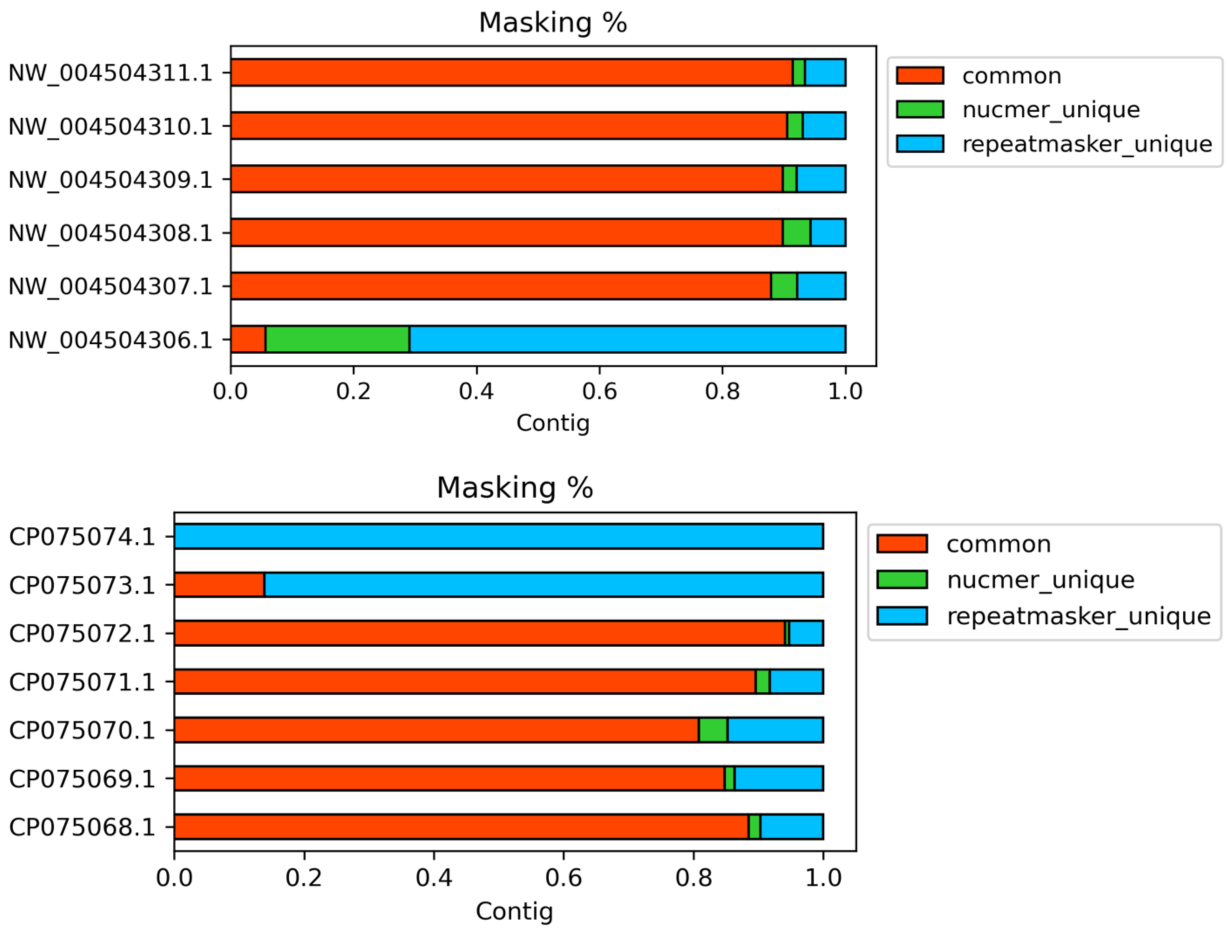

Fig. S2. Repetitive genomic regions identified by different tools. Bars indicate the percentage of the contig identified as repetitive for *C. immitis* (top) and *C. posadasii* (bottom). Colors indicate the types of masking: "common" regions identified by both NUCmer and RepeatMasker (shown in red), regions unique to NUCmer ("NUCmer_unique," shown in green), and regions unique to RepeatMasker ("repeatmasker_unique," shown in blue).
